# Direct observation of magnetoelastic coupling in a molecular spin qubit: new insights from crystal field neutron scattering data†

**DOI:** 10.1039/d2sc05797b

**Published:** 2023-03-06

**Authors:** Maja A. Dunstan, Marcus J. Giansiracusa, Michele Vonci, Simone Calvello, Dehong Yu, Alessandro Soncini, Colette Boskovic, Richard A. Mole

**Affiliations:** a School of Chemistry, The University of Melbourne Parkville Vic. 3010 Australia; b Australian Nuclear Science and Technology Organisation Locked Bag 2001 Kirrawee NSW 2232 Australia richard.mole@ansto.gov.au; c Department of Chemical Sciences, University of Padova Via Marzolo 1 35131 Padova Italy

## Abstract

Single-molecule magnets are promising candidates for data storage and quantum computing applications. A major barrier to their use is rapid magnetic relaxation and quantum decoherence due to thermal vibrations. Here we report a reanalysis of inelastic neutron scattering (INS) data of the candidate qubit Na_9_[Ho(W_5_O_18_)_2_]·35D_2_O, wherein we demonstrate for the first time that magnetic relaxation times and mechanisms can be directly observed as crystal field (CF) peak broadening in INS spectra of a lanthanoid molecular system. The magnetoelastic coupling between the lower energy CF states and phonons (lattice vibrations) is determined by the simultaneous measurement of CF excitations and the phonon density of states, encoded within the same INS experiment. This directly results in the determination of relaxation coupling pathways that occur in this molecule. Such information is invaluable for the further advancement of SMMs and to date has only been obtained from techniques performed in external magnetic fields. Additionally, we determine a relaxation rate of quantum-tunnelling of magnetisation that is consistent with previously measured EPR spectroscopy data.

## Abstract 2

Magnetoelastic coupling has been investigated using inelastic neutron scattering. This has allowed the spin–phonon coupling of the candidate qubit Na_9_[Ho(W_5_O_18_)_2_]·35D_2_O to be determined in the absence of an applied magnetic field.

## Graphical abstract

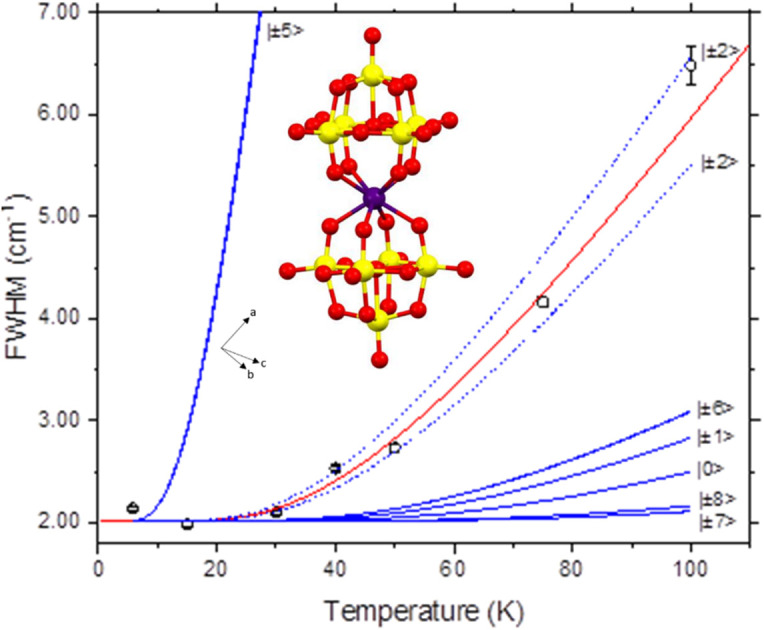

## Introduction

Single-molecule magnets (SMMs) are discrete inorganic compounds that exhibit slow relaxation of magnetisation at low temperature. The intrinsic bistability of SMMs^1–3^ has resulted in them being proposed as candidate materials for data storage,^4^ while quantum tunnelling relaxation processes give rise to applications as qubits^5^ and as components in spintronics devices.^6,7^ Application in these technologies seeks to minimise energy consumption in data storage and computing, allowing for more efficient use of finite natural resources. One of the major advantages of SMMs is that their properties can be tuned using synthetic chemistry,^8^ while the molecules themselves can be readily manipulated to allow adsorption onto surfaces^9^ and ultimately incorporation into devices.^10^ To date most candidates for quantum computing have employed dilution temperatures (typically around 10–50 mK) to minimise the noise in the system, most of which is from unwanted vibrations in the material. While there has been progress with this, including work showing silicon-based quantum dots operating above 1 K,^11^ alternatives that can address this limitation would have a significant technological advantage for use in both data storage and quantum computing. It is therefore imperative to control the vibrations and thermal relaxation in these materials.

The current best performing SMMs incorporate lanthanoid (Ln) ions as the magnetic centre of choice, and of these, compounds of the Ln(iii) ions are most prevalent. Recent advances in the field have allowed the observation of magnetic hysteresis above liquid nitrogen temperatures, a huge advance for potential use in real world devices. For a Ln(iii) ion, the magnetic properties stem from crystal field (CF) splitting of the ground ^2*S*+1^*L*_*J*_ spin–orbit term. This CF splitting can be tuned by judicious choice of ligands and is where the majority of effort in the design of high performing SMMs has focussed.^12–16^ Design guidelines based on CF criteria have been very successful, including the development of the highly axial dysprosocenium SMMs,^8,17–19^ pentagonal bipyramidal complexes with high effective energy barriers,^20–23^ and pseudo-*D*_4d_ compounds that encompass some of the first Ln-SMMs.^24,25^ Despite huge leaps in the performance of Ln-SMMs, rapid thermal relaxation and decoherence still hinder their implementation in devices.

One of the biggest challenges to the use of SMMs in devices is understanding how the magnetic properties are influenced by the external environment – in particular, the effect of vibrations on the relaxation processes.^26^ The magnetic relaxation of a SMM can occur by various means. The most commonly discussed relaxation process, Orbach type relaxation, is an over barrier relaxation pathway, which relies on thermal relaxation through excited CF states. In addition to the Arrhenius type relaxation rate observed for an Orbach type relaxation pathway, other thermal relaxation processes such as the two-phonon Raman relaxation through a “virtual” excited state or direct relaxation in an external field are possible, each with their own characteristic temperature dependence. SMMs can also relax by the non-thermally activated quantum-tunnelling of magnetisation (QTM), a through barrier relaxation process. Quantum tunnelling of magnetisation can be tuned by careful choice of Ln(iii) and symmetry, and allows for the use of SMM materials in applications such as quantum computing. Dynamic (ac) susceptibility measurements are the most widely used technique for determination of which combination of relaxation processes occur for a given compound.

To improve SMM behaviour, it is necessary to reduce the relaxation rate of all relaxation pathways. Thermally activated relaxation pathways require transfer of energy to and from an infinite “bath” of thermal energy to the spin centre, mediated *via* phonons (lattice vibrations) in the sample. The relaxation rate can be tuned by modifying not only the CF splitting (*i.e.* controlling the effective energy barrier of Orbach relaxation), but also the spin–phonon coupling by designing rigid bulky systems, minimising low energy molecular vibrations, and tuning the available phonon spectrum through modification of the lattice vibrations and symmetry. This is exemplified in the dysprosocenium ([Dy(Cp^R^)_2_]^+^, Cp^R−^ = substituted cyclopentadienyl) family of Ln-SMMs, in which compounds with various substituents on the Cp^R−^ ligands all exhibit effective energy barriers to Orbach relaxation of *Δ*_CF_ > 950 K, however they display a wide range of 100s blocking temperature (*T*_B_) values.^8,17–19^ This behaviour is ascribed to the relative efficiency of Raman relaxation, due to variation of the molecular vibrations,^19,27^ while the marked improvement over previous SMMs has been attributed to the restriction of phonons at the metal centre, arising from the bis-Cp^R−^ coordination and lack of equatorial ligands.

While spin–phonon coupling is an important effect governing the properties of SMMs,^26–29^ experimentally determining the nature of this coupling has remained a challenge, most of which has focused on using far-infrared (FIR) spectroscopy^29,30^ and computational studies.^31,32^ Inelastic neutron scattering (INS) is a technique that allows both the structural and magnetic dynamics of a material to be measured.^33^ The effects that can be probed with the technique are broad but include the measurement of phonons in single crystals,^34^ as well as the measurement of vibrational spectra and phonon density of states in a wide variety of inorganic materials.^35^ Unlike FIR and Raman spectroscopy, there are no selection rules when measuring vibrational spectra by INS, allowing observation of the entire phonon spectrum with a single technique. Additionally, an array of different magnetic excitations can also be observed, from magnons in single crystals,^34^ spinons in low dimensional quantum magnets,^36^ and the excitations of single molecule magnets.^37^ The bulk of this paper deals with the observation of CF splitting, something that has been observed in rare earth compounds almost since the inception of the technique. In recent years this has been extended to the study of single molecule magnets.^38^ The energy scale of the CF splitting of the ground state multiplet is of the same order as the energy of the neutron and selection rules mean that these transitions are possible. Subject to the selection rules of Δ*m*_*s*_ or Δ*m*_*j*_ = 0, ± 1, information about both energy and spatial dependence of an excitation can be obtained, due to measuring data in *S*(*Q*,*ω*), the double Fourier transform in both space and time. One key advantage of the technique for the study of spin–phonon coupling is that both magnetic and structural excitations can be measured simultaneously in zero applied magnetic field.

A subset of the INS technique is quasi-elastic neutron scattering or (QENS),^39^ which involves the study of the broadening of the elastic line in the INS spectrum. This portion of the spectrum contains information on relaxation dynamics of zero energy processes. It has long been used for studying the diffusive dynamics of protons^40^ and other elements,^41^ but has also been used to study magnetic dynamics including the spin state fluctuations preceding phase transitions,^42^ and the lifetime of zero-energy processes in CF split systems^43^ and molecular magnets.^44^

A key result used in the current work, is that the energy and lifetime of an excitation are not completely independent, as the lifetime of inelastic phenomena, such as CF excitations, is encoded into the inelastic spectrum as an energy broadening. Attempts have previously been made to understand spin–phonon coupling in a range of materials containing lanthanoid ions by INS,^31,45^ however the study of molecular compounds by INS is dominated by the study of CF transitions.^38,46,47^ It should also be noted that INS probes specific CF transitions, so any measured information, such as relaxation time, can be directly correlated to that transition. This is in comparison with susceptibility methods which measure an ensemble of processes resulting in loss of transition specific information.

Several other studies have been published looking at spin–phonon coupling and lifetime analysis using INS. These include a study of the 3d transition metal complexes, Co(PPh_3_)_2_X_2_ (X = Cl, Br, I), which demonstrated that INS is a unique zero field technique that can identify both magnetic excitations and phonons in a molecular magnet.^48^ This study relied on analysis of the FIR spectroscopy with an applied magnetic field to identify avoided crossings and thus determine the spin–phonon coupling. Other studies of SMMs using 4D INS spectroscopy have additionally shown that under barrier relaxation *via* anharmonic phonons and coupling with the acoustic phonons are important pathways for relaxation.^28,48,49^ In addition, INS data contain information about lifetimes of excitations, and has previously been used to study relaxation rates in a {TbCu} single-molecule magnet.^44^

In order to design SMMs for use in real world applications, not only are the electronic properties important, but clearly the vibrational properties are too, studies of which are still lacking. Here we discuss the candidate qubit [Ho(W_5_O_18_)_2_]^9−^,^50^ a SMM shown to have so-called atomic clock transitions^51^ that protect the molecule from dipolar relaxation at key external fields. However, thermal effects remain a known issue for this compound. The present work was inspired by a recent report of a spectroscopic analysis^52^ of [Ho(W_5_O_18_)_2_]^9−^, which examined FIR spectra, both with and without an applied magnetic field, complemented by *ab initio* electronic structure and density functional theory (DFT) calculations. This work provided evidence for coupling between the CF excitations and the phonons. We have now gone back to our original INS data^53^ to examine the spectroscopic signatures of spin–phonon coupling. We report an alternative analysis of the dataset, presenting both the generalised density of states and the temperature dependence of the lifetime of the CF excitations. Specifically, we demonstrate that spin–phonon coupling information is encoded in the zero field, temperature-dependent, INS spectra from a typical CF investigation. Therefore, from a typical CF investigation with INS, additional information can be extracted to inform SMM design properties. This alternate analysis of existing data is fundamentally similar to the distribution of relaxation times in a Debye model fitting correlating to the thermal ellipsoids of single-crystal XRD data, or the use of X-ray/neutron diffraction to extract spin density information about a magnetic system. These two examples have provided additional fundamental understanding of experimentally observable relaxation dynamics of molecular magnets. We hope this study will inform the use of INS as a tool for the observation of spin–phonon coupling in SMMs, with the ultimate aim of guiding the design of SMMs for use in real-world devices.

## Experimental

### Synthesis

The synthesis and characterisation details of the 3 g sample Na_9_[Ho(W_5_O_18_)_2_]·35D_2_O were reported previously.^53^ The sample was deuterated to reduce the significant incoherent scattering from ^1^H in the sample. An estimated 80% deuteration was achieved.

### Inelastic neutron scattering

The INS measurements are as previously reported,^53^ and were carried out using the Pelican time-of-flight spectrometer at the Australian Nuclear Science and Technology Organisation facilities at Lucas Heights.^54,55^ All manipulations and sample fitting were carried out using the LAMP software.^56^ Data were collected with *λ* = 4.69 Å with a higher resolution of Δ*E* = 1.09 cm^−1^ at the elastic line, as well as with *λ* = 2.35 Å (with a resolution of Δ*E* = 6.86 cm^−1^ at the elastic line) to allow measurement of CF transitions as cold transitions. The measurements were all taken with a Fermi chopper frequency of 100 Hz and the resolution accounted for as described in ref. 54 and 55. The generalised phonon density of states was calculated using the neutron energy gain spectrum from the 4.69 Å data, which has an appreciable population of phonon modes at 300 K.

### Crystal field splitting

The CF splitting used in the analysis of this work comes from the previously reported *ab initio* electronic structure calculations.^53^ The CF energies and compositions used here agree with the experimental fits of INS data,^53^ as well as the recently reported CF splitting by Blockmon and Ullah *et al.* (Fig. S1†).^52^

### Structure

**Fig. 1:**
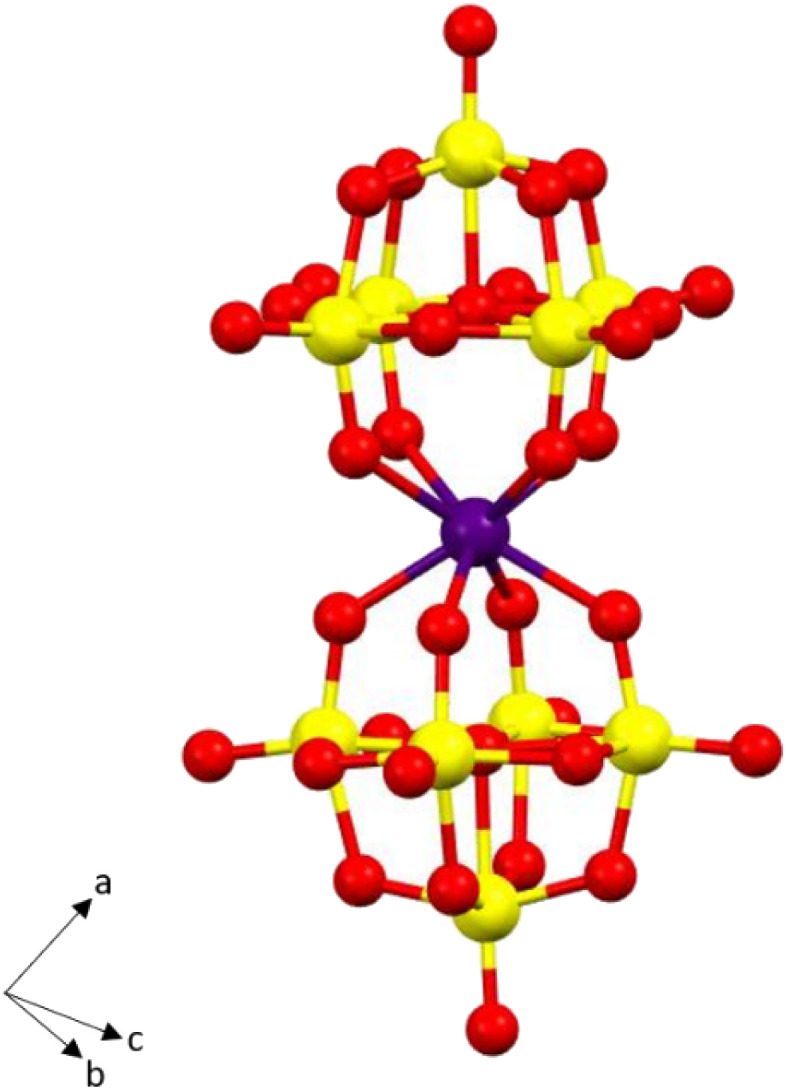
Ball-and-stick representations of the [Ho(W_5_O_18_)_2_]^9−^ polyanion, water and sodium ions excluded for clarity. Colour scheme Ho: purple, W: yellow, O: red.

The single crystal X-ray structure of the nondeuterated analogue Na_9_[Ho(W_5_O_18_)_2_]·35H_2_O has been reported previously.^53^ Single-crystal X-ray structural analyses indicate that the compound is comprised of a unique [Ho(W_5_O_18_)_2_]^9−^ polyanion surrounded by a network of water-coordinated Na^+^ cations (Fig. 1). The Ho(iii) ion is octacoordinated by the four oxygen anions provided by each of the {W_5_O_18_} metalloligands in a slightly distorted square antiprismatic geometry providing a *D*_4d_ pseudosymmetry at the Ho centre.

## Results

### Inelastic neutron scattering

The inelastic neutron scattering data of the deuterated Na_9_[Ho(W_5_O_18_)_2_]·35D_2_O were analysed in three different ways: analysis of the phonon modes from the generalised phonon density of states, a lifetime analysis of the crystal field excitations, and analysis of the quasi-elastic neutron scattering (QENS) spectra.

**Fig. 2:**
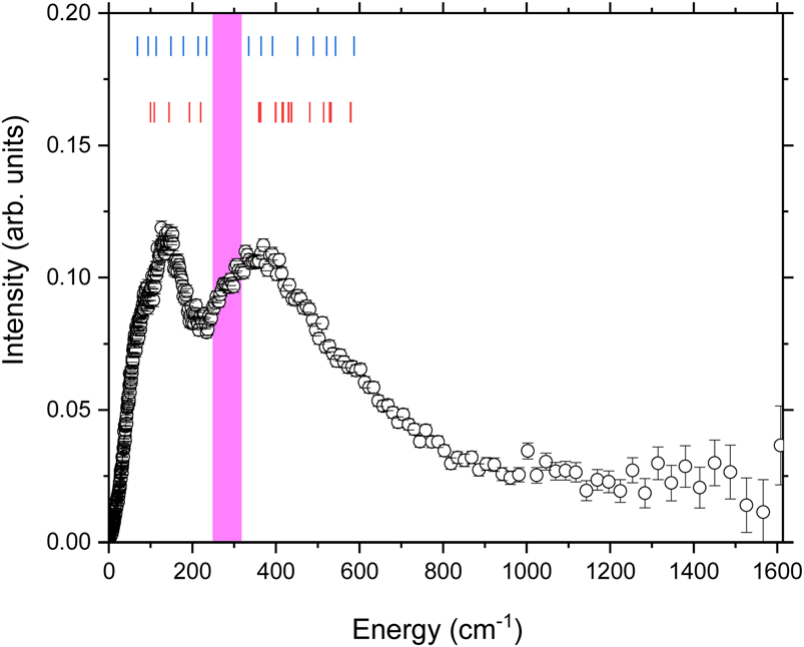
The generalised phonon density of states at 300 K of Na_9_[Ho(W_5_O_18_)_2_]·35D_2_O determined using the method described in the text. The blue tick marks represent the modes observed by FIR on the same compound.^52^ The red tick marks the vibrational modes calculated using DFT^52^ for the anion [Ho(W_5_O_18_)_2_]^9−^. The pink highlighted region is discussed in the text.

Firstly, the generalised density of states (GDOS) was determined to allow identification of major phonon modes in the sample. To do this, data collected at 75 K and above where there is a significant population of phonon modes, were converted to the two-dimensional generalised density of states (*g*(*Q*,*ω*)) using the following formula:1
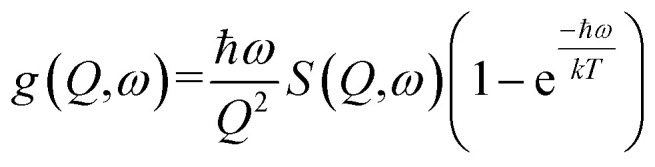
which corrects the scattering function *S*(*Q*,*ω*) for temperature by the Bose factor for phonons of energy *E* = *ħω*. The experimental GDOS, *g*(*ω*), for the 300 K data is shown in Fig. 2. The following features can be observed: a maximum at 138.7(8) cm^−1^ and an additional maximum at 358.1(2) cm^−1^, which were determined by fitting to a Gaussian in a restricted range about each maximum. There is then a broad tail stretching to 1600 cm^−1^. The observed maxima agree well with the two major clusters of vibrational modes determined from FIR and DFT.^52^

**Fig. 3:**
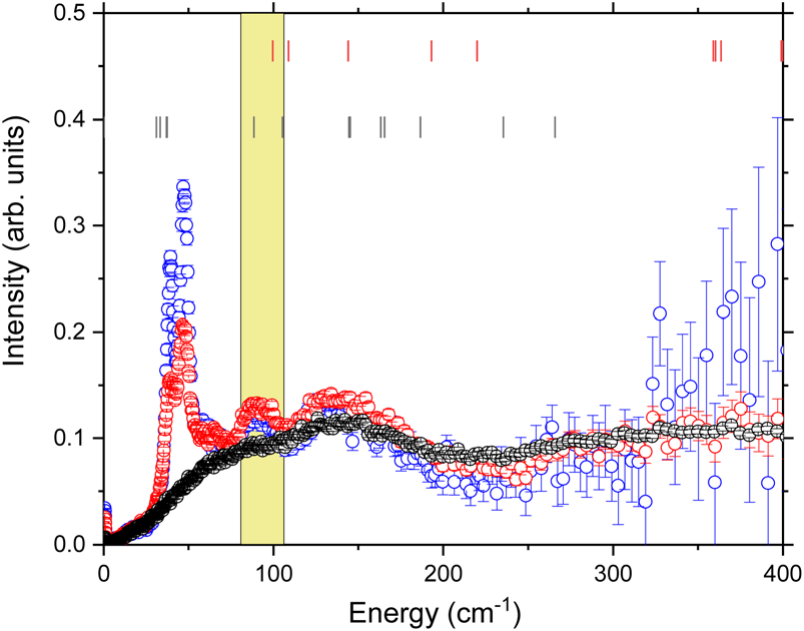
Temperature dependence of the phonon density of states, obtained using 4.69 Å data, focussed on the low energy region. Black 300 K, red 100 K, blue 75 K. Red ticks are the theoretical mode frequencies from ref. 52, black ticks are the theoretical crystal field levels from ref. 53. The yellow highlighted region is the energy range determined by the magnetoelastic model discussed in the text.

The temperature dependence of the lower energy (*E* < 400 cm^−1^) GDOS is shown in Fig. 3. The first features that are apparent in the 75 and 100 K data are two sharp peaks at 40.62(2) cm^−1^ and 48.96(2) cm^−1^. These can be assigned as the reported CF transitions in this compound. Phonons and crystal field excitations show a very different temperature dependence when corrected by the Bose factor. The magnetic crystal field excitations originate from unpaired electrons in a sample, so are fermions, while phonons are bosons. Additionally, upon cooling to 75 K from 300 K, the portion of the spectrum observed is reduced to energies below approximately 400 cm^−1^ due to the reduced thermal population, but the peaks that remain are clearer due to the reduced Debye–Waller factor.^35^ Of the two major phononic features observed at 300 K, only the 138.7(8) cm^−1^ peak is now observed, but this is now resolved into two clear peaks at 90.9(4) cm^−1^ and 138.7(8) cm^−1^.

### Crystal field lifetime analysis

As stated, two crystal field excitations have been reported for this compound (Fig. S1†).^53^ These correspond to the transitions between the ground *m*_*j*_ = |± 4> pseudodoublet and the two lowest lying excited pseudodoublets, *m*_*j*_ = |± 3> (*E* = 39.80 cm^−1^) and *m*_*j*_ = |±5> (*E* = 48.28 cm^−1^) as fit previously from the full *Q* integration of the *S*(*Q*,*ω*) data. Here we discuss the fitting procedure to extract lifetime information from these two observed transitions.

The methodology for extracting lifetimes from peak broadening in INS is well known and has been reported in the literature for many years,^58^ however, has not yet been applied to SMM compounds. Instead of fitting the crystal field excitation to a Dirac delta function convoluted with the instrument resolution, the data are fit to a Lorentzian function convoluted with the instrument resolution. The variation in width of the Lorentzian is then proportional to the lifetime of the crystal field excitation.

**Fig. 4:**
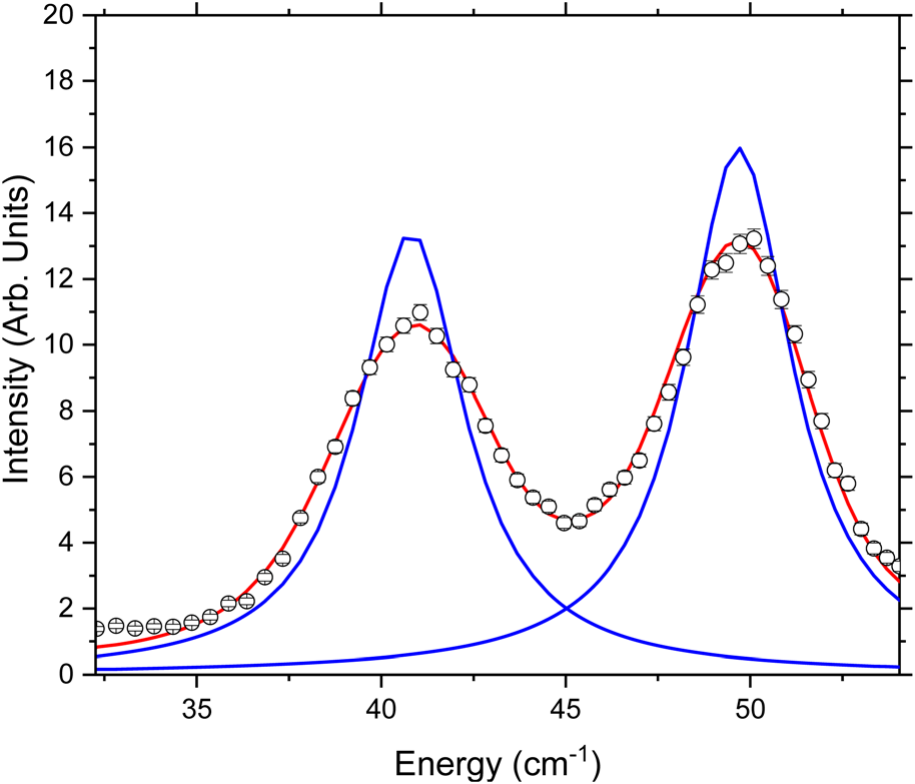
Example fit of *S*(*ω*) measured using 2.35 Å neutrons at 30 K. The fits shown here are to two Lorentzians constrained to have the same width and convoluted with the experimentally determined resolution function. The black circles are the measured data points, the blue line is the individual Lorentzian contributions, and the red line is the total fit after convolution with the resolution function.

The following fits were performed on the data recorded with *λ*/2 = 2.35 Å at all temperatures (Fig. S2–S3†). The data were integrated over all measured *Q* and the two peaks at *E* = 39.80 cm^−1^ and 48.28 cm^−1^ were fit to two separate Lorentzians convoluted with the experimental resolution function determined by measuring a vanadium standard. Two separate fits were performed: model 1 with the Full Width at Half Maximum (FWHM) allowed to refine freely for both Lorentzians (Fig. S2†); and model 2 with the two Lorentzians constrained to have the same value (Fig. 4 and S3†). Both models give good agreement with the data, although the error is lower on model 2, so these fits were used for further analysis. The implications of this are addressed in the Discussion section.

### Quasi-elastic neutron scattering

**Fig. 5:**
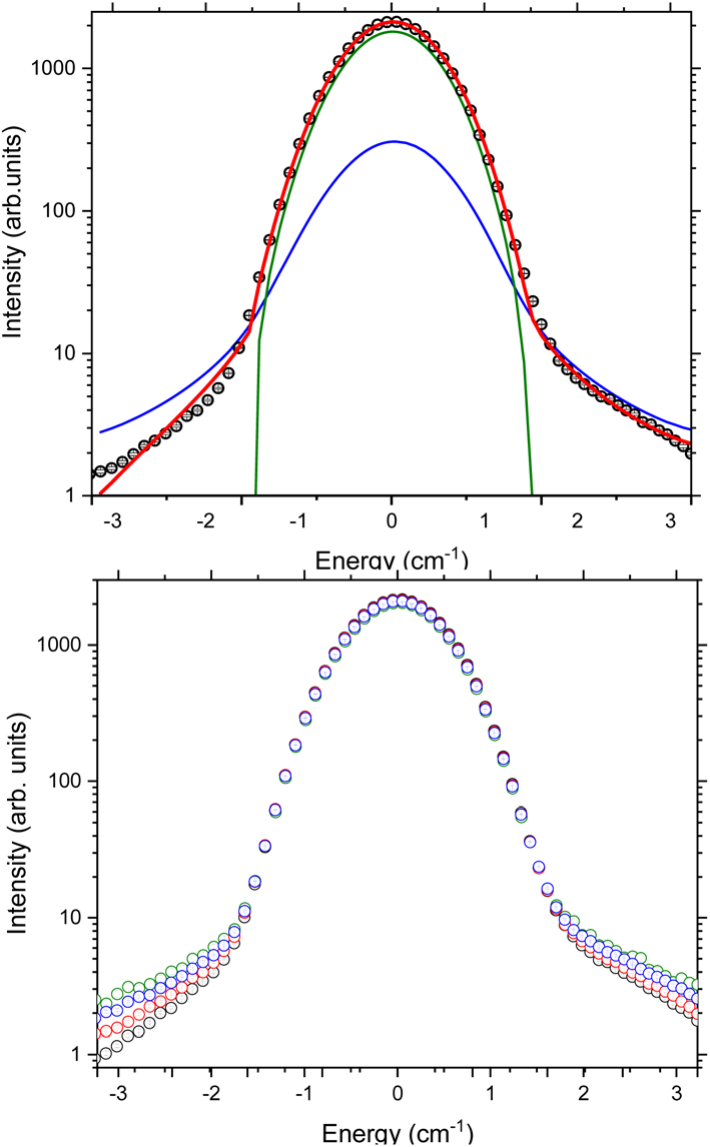
(Top) Fit to the quasi-elastic portion of the spectrum collected with 4.69 Å neutrons measured at 50 K (data shown as black symbols). The red line is the total fit, the blue line is the Lorentzian component convoluted with the experimental resolution function and the green line is a delta peak convoluted with the experimental resolution function. (Bottom) Temperature dependence of *S*(*ω*) measured using 4.69 Å neutrons. Colour scheme, black datapoints 1.5 K, red 50 K, blue 90 K, green 150 K.

Finally, we analysed the quasi-elastic neutron scattering (QENS) portion of the spectrum. This was done in a similar manner to the CF lifetime analysis, using the data collected with *λ* = 4.69 Å to give higher resolution near the elastic line. The data were converted to *S*(*Q*,*ω*) and then integrated over all *Q*. These data show an elastic line broadening greater than that observed in the vanadium standard (Fig. 5). To quantify this peak broadening the data were fit to a delta peak and a Lorentzian, both constrained to have the same centre (nominally 0 cm^−1^) and a linear background term (Fig. 5 top). The fit was also constrained to obey detailed balance,^34^ which relates the intensity of magnetic scattering on the neutron energy loss side to that on the neutron energy gain side:

2 *S*(*Q*,−*ω*) = *S*(*Q*, *ω*)e^(−*ħω*/*kT*)^.

To allow for the change in background with temperature due to population of phonon modes, and the proximity of the low-lying crystal field excitations, the fit was constrained to be between ±3.2 cm^−1^. The temperature dependence of the FWHM of the Lorentzian contribution was then determined between 1.5 K and 150 K. While the data clearly show changes as a function of temperature, the Lorentzian component of the elastic line has a FWHM that does not show a temperature dependence and is 0.32 cm^−1^ within measurement error at all temperatures.

## Discussion

Here we first consider the temperature dependence of the peak widths of the crystal field excitations. Inelastic neutron scattering allows us to measure not just the crystal field excitations and the phonons, but also the lifetimes of the excitations. In an INS experiment, the measured quantity *S*(*Q*,*ω*) is given by:3
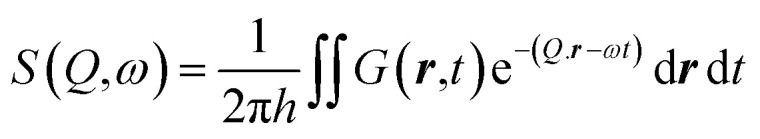
Here *G*(***r***,*t*) is the dynamic response function, which provides information on the position of atoms and unpaired electrons as a function of time.^57^ Experimental data are typically recorded as the double Fourier transform *S*(*Q*,*ω*). The transform from time to energy is the reason we can use this technique to determine the energy scale of the system – this is the conventional analysis of crystal field excitations. However, as the function is clearly dependent on time, the lifetime of any excitation is also encoded into the data. This is a well-known phenomenon and has been utilised widely in condensed matter physics to study the lifetime of CF excitations,^58^ spin-waves,^59^ and phonons.^60^ In this paper we focus solely on the crystal field lifetimes.

### Magneto-elastic coupling

There are several relaxation pathways in magnetic materials, and each has a well-known temperature dependence. Examples include relaxation *via* phonons, such as an exponential growth *via* an Orbach mechanism,^61^*via* conduction electrons which demonstrate the so-called Korringa law^62^ (which is a power law dependence), or as quantum tunnelling of magnetisation, which is temperature independent. In the case of SMMs in the crystalline state, as electrical insulators, the only known relaxation mechanisms are *via* coupling to phonons and any quantum relaxation. A magnetoelastic coupling model that results in a single phonon type relaxation has previously been proposed by Lovesey and Staub,^58^ which we use here to analyse our data. The model requires a magnetoelastic operator to be introduced which has the following properties:4
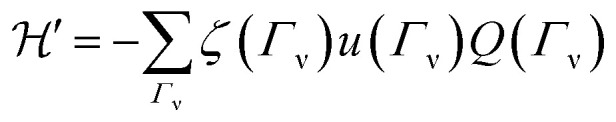
where *ζ*(*Γ*_ν_) is a magnetoelastic coupling parameter, *u* (*Γ*_ν_) is a normal mode that transforms according to the representation *Γ*_ν_, and *Q* (*Γ*_ν_) is a quadrupole operator used to represent the 4f valence electrons of the Ho(iii) ion.

**Fig. 6:**
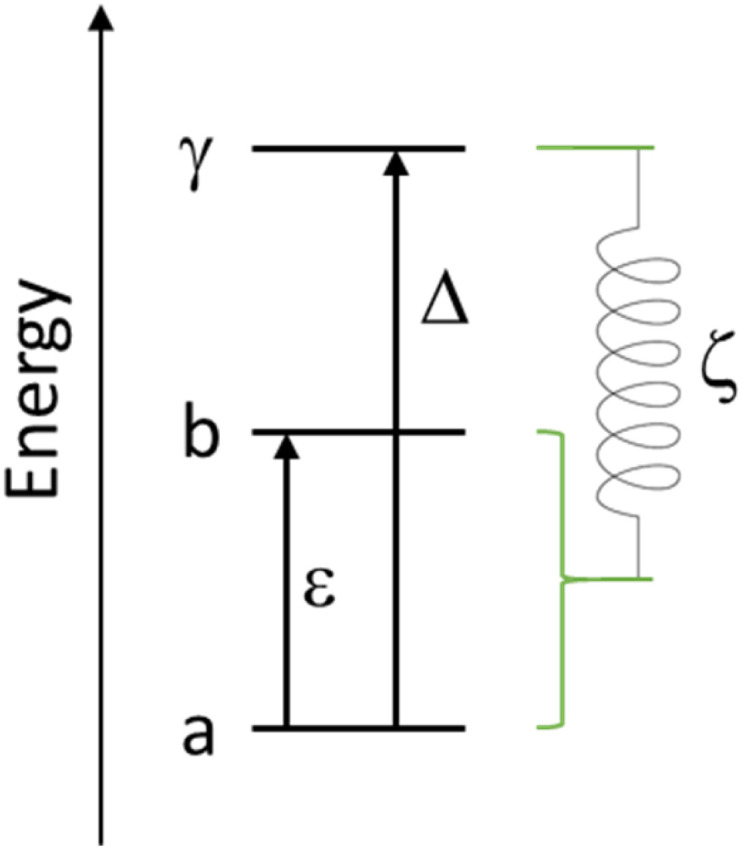
Schematic showing the simple energy level diagram used to construct eqn (5). The magnetoelastic coupling is shown as a spring linking the ground and excited crystal field states to a higher crystal field state, the parameter *ζ* has dimensions of energy and length.

A three-state model is constructed to model the lifetime of an excited state at *E* = *ε* relative to the ground state (Fig. 6). A third state at an energy *Δ* relative to the ground state is then coupled to each of these states *via* the magnetoelastic coupling parameter defined previously (eqn (4)). The normal modes are incorporated into the model *via* the phonon density of states. This gives rise to the result that the FWHM with temperature of the excitation from the ground state to the state at *E* = *ε*, *Γ*(*T*), is given by5
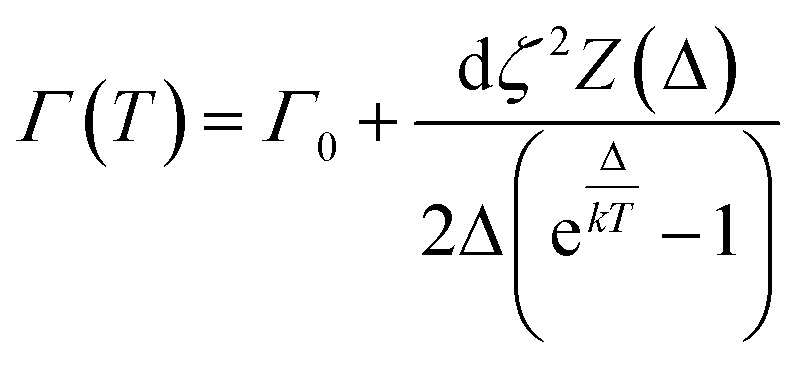
where *Z*(*Δ*) is the phonon density of states *at ħω* = *Δ*, *Γ*_0_ is the residual FWHM, and *d* is a constant that incorporates the magneto-elastic coupling of the ground state and the excited state.

The relationship between the theoretical phonon density of states and the measured phonon density of states is described in the ESI,† as is a more detailed description of the three-state model and its assumptions derived in ref. 58.

**Fig. 7:**
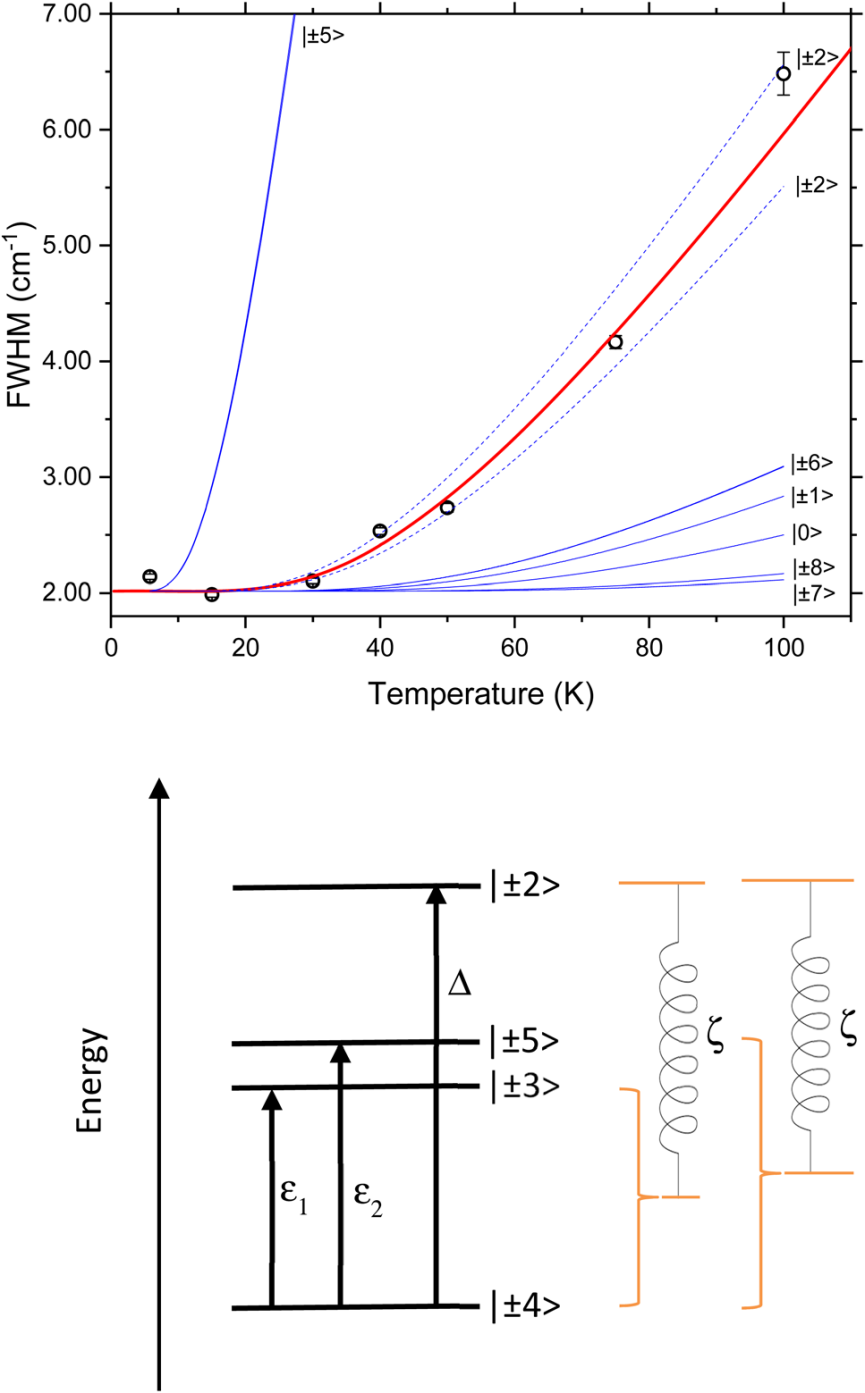
(Top) Temperature dependence of the FWHM of the Lorentzian component of the fit to *S*(*Q*) shown in Fig. 4 and described in the text. The red line is a fit to the Lovesey and Staub model (eqn (5)). The blue lines are simulations of the same model with the same *d* and *Γ*_0_ and the value of *Δ* set to be the possible excited state crystal field levels predicted by electronic structure calculations.^53^ The dashed blue lines are those corresponding *Δ* having the calculated values for the *m*_*j*_ = |±2> pseudo doublet. (Bottom) Schematic showing the magnetoelastic coupling model as applied to [Ho(W_5_O_18_)_2_]^9−^ with CF states as determined by INS and calculations.^53^

The temperature dependence of the experimental FWHM of the crystal field excitations determined by INS using fitting model 2 (Fig. 7) shows a clear temperature dependence, and was fit to eqn (5), with the numerator combined to give a refinable constant. In doing this we are making the assumption that *ε*_1_ = 39.80 cm^−1^ and *ε*_2_ = 48.28 cm^−1^ are coupled to the same excited crystal field level *Δ*. The best fit to the data gives *Δ* = 94.5 (12.0) cm^−1^ and is shown by the solid red line in the figure. The same parameters also demonstrate a good description of the FWHM determined using fitting model 1 (Fig. S4†) with only the residual *Γ*_0_ varying. The variation in *Γ*_0_ is expected, as this part of the magnetoelastic coupling model^58^ is dependent on *ε* and the nature of the ground state (*a*) and first excited state (*b*). To be an allowed magnetoelastic coupling, the final requirement is that *Δ* must be in a region with a vibrational density of states. The range of values of *Δ* are highlighted in yellow in Fig. 3 and clearly coincide with a peak in the measured phonon density of states *g*(*ω* = *Δ* = 94.5 cm^−1^). The highlighted region also demonstrates that this is the same energy as crystal field states predicted by *ab initio* electronic structure calculations verified by previous INS measurements.^53^ These calculations predict states of predominantly *m*_*j*_ = |±2> pseudodoublet at 88.47 cm^−1^ and 105.33 cm^−1^, near the fitted value of *Δ*.

If we replace the fitted *Δ* value in eqn (5), assuming now that *Δ* is equal to each of the *ab initio* calculated energies for the *m*_*j*_ = |±2> pseudodoublets, we calculate the dashed blue lines in Fig. 7, highlighting that our fit value lies between these states. The solid blue lines in Fig. 7 are equivalent simulations using *Δ* values for the other low energy CF levels previously calculated using *ab initio* methods, demonstrating that when the temperature dependence of the FWHM is calculated with these energies, it does not match with the experimentally observed magnetoelastic coupling to the *m*_*j*_ = |± 3> and *m*_*j*_ = |± 5> states. These fits and simulations lead us to conclude that the relevant model is the one shown on the lower panel of Fig. 7.

We now consider whether the same value of *Δ* should be observed for both CF excitations. Following the model of Lovesey and Staub, they introduce the magnetoelastic coupling parameter with the caveat that the CF level and the vibration have to transform with the same irreducible representation and thus have the same symmetry. If the two CF levels with *m*_*j*_ = |± 3> and *m*_*j*_ = |± 5> have different symmetries, they should not have the same value of *Δ* as they should not both couple to *m*_*j*_ = |± 2>. While the local symmetry of the Ho(iii) ion can be approximated as *D*_4d_, this is not accurate, and the point group is strictly *C*_s_. In this case, all CF states and phonons have the same symmetry and can mix as there is only one irreducible representation. Thus, having both the low energy CF states couple to the same excited state is reasonable within this model. It is also noted that with this argument it should be possible to observe coupling between *ε*_1_ and *ε*_2_, however there is no sizeable phonon density of states *g*(*ω*) for *E* = *ε*_2_ = 48.28 cm^−1^ so magnetoelastic coupling would not be allowed *via* the currently proposed mechanism.

Next, we should consider the contribution from the phonon density of states *g*(*Δ*). We compare the measured density of states to the FIR measurements of Blockmon and Ullah *et al.*^52^ The FIR vibrational modes are shown as blue tick marks in Fig. 2. As expected, the key features of the spectrum are replicated between the INS GDOS and the FIR data, though the relative intensities are different due to the difference in scattering cross sections and selection rules between the two techniques. Both datasets can be summarised as having broad groups of excitations centred around 139 cm^−1^ and 358 cm^−1^.

The data were also compared to the lattice dynamics calculations of Blockmon and Ullah *et al.*,^52^ which are shown as red tick marks in Fig. 2. These also reflect the general form of the data with two clusters of excitations in the same energy regimes. One of the main conclusions of the work of Blockmon and Ullah *et al.* is that sparse spectra are key to minimising spin–phonon coupling and that the weak spin–phonon interactions for this compound are attributed to a calculated gap in the phonon spectrum. While the general form of the INS spectra is the same as that for the FIR and DFT, we do however note that there is significant intensity observed in the phonon density of states from 250 cm^−1^ to 314 cm^−1^. This region is highlighted in pink in Fig. 2. These modes are likely not predicted in the DFT calculations because the calculations were done considering only the bare anion. The INS measurements were performed on the real system where there are 9 sodium cations and 35 lattice water molecules – these will also add to the spectroscopic signature for the sample. A survey of the literature reveals that nearly all ices,^63^ gas hydrates,^64^ and clays^65^ that have been extensively studied using neutron vibrational spectroscopy have key spectroscopic signatures from water molecules in this energy range. The phonon density of states of 12-tungstophosphoric acid hexahydrate has also been reported and shows intensity in this region which is assigned to vibrational modes of the water.^66^ Given the good correspondence between the FIR and DFT calculations of Blockmon and Ullah *et al.*,^52^ it is reasonable to conclude that the remaining modes are from the water molecules. This might suggest the need to consider the whole system and not use the [Ho(W_5_O_18_)_2_]^9−^ anion, as the possibility exists that the crystal field excitations of the Ho ion could couple to the “bath” of vibrations of similar energy from the water molecules in the lattice and form another pathway for vibrational relaxation. However, the current work demonstrates that the major magnetoelastic coupling mechanism is due to phonon modes which strongly couple to the Ho(iii) centre and there is no evidence of additional higher energy terms, which could occur due to coupling from the “bath” of water excitations or other vibrational modes from the poly oxo tungstate ligand.

Looking more closely at the low energy portion of the spectrum (Fig. 3) the following modes assigned by DFT can be identified in the neutron scattering dataset. The asymmetric cage tilts of the {HoO_8_} are predicted from DFT to be at 99.6 cm^−1^ and 108.8 cm^−1^. These correspond to the observed peak at 90.9(4) cm^−1^. Stretching of the (WO_5_)_2_ units are theoretically predicted at 144 cm^−1^ and observed at 138.7(8) cm^−1^. Overall there is a good correspondence between the lattice dynamics and the observed phonon spectrum.

We can then bring the information from the GDOS and lifetime analysis together. Fitting the width of the crystal field excitations to the magnetoelastic coupling model predicted a crystal field excitation with an energy of *Δ* = 94.5(12) cm^−1^ – this energy range is highlighted in yellow in Fig. 3. The model of Lovesey and Staub for magnetoelastic coupling relies on there being a contribution from the phonon density of states at the final CF energy *Δ*. The theoretical phonon density of states has shown that there is a phonon at 99.6 cm^−1^, which corresponds to an asymmetric cage tilt as assigned previously. The fact that the asymmetric cage vibration of the {HoO_8_} is responsible is a logical conclusion as this will give rise to the largest shift away from the idealised *D*_4d_ symmetry of the rare earth ion. Further, it is also logical that this mode causes relaxation as this directly involves the Ho(iii) centre.

We conclude from this single temperature dependent dataset that magnetoelastic coupling occurs in Na_9_[Ho(W_5_O_18_)_2_]·35D_2_O and results in a thermally activated relaxation for both the *m*_*j*_ = |±3> and *m*_*j*_ = |±5> pseudodoublets by coupling to the *m*_*j*_ = |±2> with the asymmetric cage tilts of the {HoO_8_}. Such a mechanism is likely the origin of the strongly temperature dependent coherent time *T*_2_ in the reported atomic clock transitions.^51^ The reported FIR study concluded that there is coupling of the same antisymmetric {HoO_8_} cage tilt to the *m*_*j*_ = |±5> state (and possibly the *m*_*j*_ = |±2>), as well as the higher energy *m*_*j*_ = |±7> mode coupling to various rocking and bending modes. We note that the FIR measurements are taken in large magnetic fields (5–35 T) while the measurements reported here are done in zero applied field, and as such are not directly comparable.

It is of note that the electronic structure calculations predict a series of CF excitations within the ground state multiplet up to 263 cm^−1^ and that there is also significant intensity in the phonon density of states in this entire region. As such, from eqn (5), any mode could in theory result in magnetoelastic coupling, including the significant vibrational bath due to the water molecules. As we observe no evidence of magnetoelastic coupling with any of these modes in the INS data this has significant implications for any potential application. If one was to construct a device out of these molecules, the pathway for magnetic relaxation in zero field appears to involve only the coordination sphere of the central rare earth ion. The vibrational bath from the medium it is held within or the surface it is attached to are apparently independent and will not need to be taken into account. Further, if the surface the molecule is bound to could be designed or optimised to either minimise this asymmetric stretch so that its intensity *g*(*Δ*) is significantly reduced or shifts by even a few 10's of wavenumbers, then this relaxation pathway may become negligible.

### Quasi-elastic neutron scattering analysis

To complete this reanalysis, we finally consider the broadening of the quasi-elastic neutron scattering (QENS) signal. As this persists at low temperatures, this is unlikely to come from the self-diffusion of water. Such water typically only shows dynamic behaviour above temperatures of around 150 K and even then, the dynamics are often too slow for a time-of-flight spectrometer such as Pelican and would typically require a high-resolution backscattering spectrometer.^67^ We consider two pathways for magnetic relaxation, either quantum tunnelling or Orbach relaxation process from the ground doublet. We will discuss both here briefly.

During Orbach relaxation a phonon is adsorbed to excite the spin system to an energy level *Δ*_CF_ above the ground state, followed by emission of a phonon to return the system back to the ground state. This process is known^43^ to give rise to a temperature dependence of the QENS line width given by:6
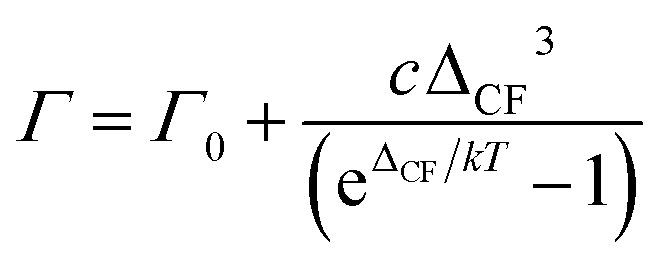
where *c* is a factor that considers the coupling of the CF ground state with the CF excited state and *Γ*_0_ is as defined in eqn (5).

For Na_9_[Ho(W_5_O_18_)_2_]·35D_2_O, we do not observe a temperature dependence to the quasi-elastic line width (data measured up to 100 K). While this does not preclude Orbach relaxation, it does put a lower limit on *Δ*_CF_. Eqn (6) would show no temperature dependence below 100 K if *Δ*_CF_ was approximately 500 cm^−1^ or higher, for lower *Δ*_CF_ values a temperature dependence would be observed. Electronic structure calculations and CF fitting^53^ applied to the lowest lying levels of the multiplet have shown that the crystal field splitting within the ground spin–orbit multiplet is only to a maximum of 263 cm^−1^. While it is unusual for a Ln-SMM to relax over the top of the energy barrier, even this is significantly lower than the *Δ*_CF_ required by a hypothetical Orbach process. Thus, we conclude that an Orbach process is not the origin of the QENS signal in Na_9_[Ho(W_5_O_18_)_2_]·35D_2_O.

Secondly, we consider quantum tunnelling of magnetisation. This is the more likely candidate process as it is temperature independent. From fits of the QENS signal, a FWHM of ∼0.3 cm^−1^ is obtained. Previous EPR measurements reported for this compound determined atomic clock transitions with a tunnelling gap of 9.7 GHz (0.32 cm^−1^) observed at magnetic field positions of 23.6, 70.9, 118.1, and 165.4 mT, which is in excellent agreement with the FWHM observed here in zero-field. This matching of the EPR transition along with the temperature independence of the FWHM is an interesting observation and provides support for the QENS originating from the QTM relaxation. However, without the ability to perturb the system in some other way (*e.g.* with a magnetic field) we cannot unambiguously assign this process.

While the analysis presented here is focussed on the presence of magnetoelastic coupling, the reanalysis of the INS dataset shows the presence of residual relaxation in the fits of both the CF excitations and the QENS data. These experiments are inherently sensitive to these additional contributions to the relaxation. Dipolar relaxation and the minimisation of these effects at the atomic clock transitions has previously been reported.^51^ Further work on this material could probe the atomic clock phenomenon by establishing how these residual linewidths are affected by an applied magnetic field (requiring fields of a few hundred Oe). The QENS signal would also be sensitive to an external field, which would allow multiple contributions to the relaxation to be observed in one experiment. Nevertheless, the extraction of this additional information has allowed the observation of relaxation dynamics in a molecular qubit from INS data which was originally performed purely for the investigation of CF energies.

Finally, we should discuss these observed timescales with respect to previously published data for Na_9_[Ho(W_5_O_18_)_2_]·35H_2_O. The published ac susceptibility^50,53^ shows an out-of-phase component to the susceptibility at the edge of the observable frequency window. As such it is not possible to extract the relaxation times nor mechanisms at these low temperatures. Magnetic relaxation rates have also been observed with EPR, which reveal microsecond *T*_1_ times, however, these are again observed only at low temperatures. The current data indicate that fast magnetoelastic coupling occurs at higher temperatures (the onset of this relaxation is *ca.* 30 K) which is far beyond the temperature window where an EPR echo or ac susceptibility response can be observed for this complex.

## Conclusions

The reanalysis of the INS data of Na_9_[Ho(W_5_O_18_)_2_]·35D_2_O has shown that there is significant further information encoded in the INS spectra, aside from the previously reported crystal field transitions. The same INS measurement allows the observation of the vibrational density of states and the temperature dependence of the lifetimes of the CF excitations, which have direct implications for the magnetic relaxation and quantum coherence in this material. Previously published DFT calculations have identified the low energy vibrational modes. Analysis of these data within the magnetoelastic coupling model indicates the modes that are responsible for magnetoelastic coupling (and thus magnetic relaxation). We have concluded that we observe magnetoelastic coupling between the *m*_*j*_ = |±2> state and the {HoO_8_} asymmetric stretch, which facilitates excitation from the ground *m*_*j*_ = |±4> state.

While this is a broadly similar conclusion to that of Blockmon and Ullah *et al.*,^52^ the measurement in zero magnetic field, available using INS, are in conditions closer to those required for the atomic clock transitions that make this complex of fundamental interest. Additionally, zero-field measurements are of direct relevance to any zero-field SMM. Such a magnetoelastic coupling mechanism would cause thermal decoherence and limit the use of the molecular qubit, as these processes would lead to noise and errors in any functional quantum computer. The lack of observation of coupling to other vibrational modes is encouraging for the design of devices, supporting the conclusion of Blockmon and Ullah *et al.*, which suggests that only modes involving the central rare earth ion need to be tuned. As such adsorption onto a surface may well provide a significant enough perturbation of this vibrational mode to remove the thermal decoherence pathway. In such a scenario, we anticipate that the surface modes will not couple to the lanthanoid ion in a manner similar to that observed for the vibrations from water in this work.

Inelastic neutron scattering requires access to large and expensive specialised research infrastructure, and therefore the ability to glean additional information from any single experiment is invaluable. We have revealed valuable insight into the relaxation dynamics in these materials, which can be obtained through simultaneous measurement of CF excitations, vibrational spectra, and lifetimes. A similar analysis of INS data could be readily applied to other candidate SMMs or molecular qubits, and thus play an important role in the design of materials for devices. Additional INS experiments using both applied magnetic fields and variation of temperature are logical extensions to this work. Similarly, the use of polarised neutrons^54,68^ could allow the magnetic and vibrational components to be measured separately and determine the extent of any mixing. The interplay of vibrational and magnetic excitations has direct implications for the use of SMM compounds in data storage and quantum computing applications. Here we have demonstrated that INS spectroscopy allows direct measurement of this, as applied to a molecular qubit.

## Data availability

The inelastic neutron scattering data presented in this work is archived by the Australian Nuclear Science and Technology Organisation. This data is freely available and can be requested by contacting enquiries@ansto.gov.au or by contacting the corresponding author.

## Author contributions

RAM conceived the idea, MV prepared and characterised the sample, MV, MJG, DY and RAM carried out the neutron scattering measurements, RAM and MAD performed the fitting and data analysis, SC and AS assisted with the theoretical basis for the work, CB provided project support and personnel supervision. RAM, MAD and MJG wrote the manuscript with contributions from all authors.

## Conflicts of interest

There are no conflicts to declare.

## Supplementary Material

SC-014-D2SC05797B-s001
