# Supplementary material for: Direct observation of magnetoelastic coupling in a molecular spin qubit: new insights from crystal field neutron scattering data
Source: Chem Sci. 2023 Mar 6;14(15):3990–4001. doi: 10.1039/d2sc05797b (PMC10094165; doi:10.1039/d2sc05797b)
Supplement: SC-014-D2SC05797B-s001 [file SC-014-D2SC05797B-s001.pdf]

Electronic Supplementary Material (ESI) for Chemical Science.
This journal is © The Royal Society of Chemistry 2023

Supplementary information for:

Direct observation of magnetoelastic coupling in a molecular spin qubit: new insights from crystal field neutron scattering data

Maja A. Dunstan, Marcus J. Giansiracusa, Michele Vonci, Simone Calvello, Dehong Yu, Alessandro Soncini, Colette Boskovic, Richard A. Mole

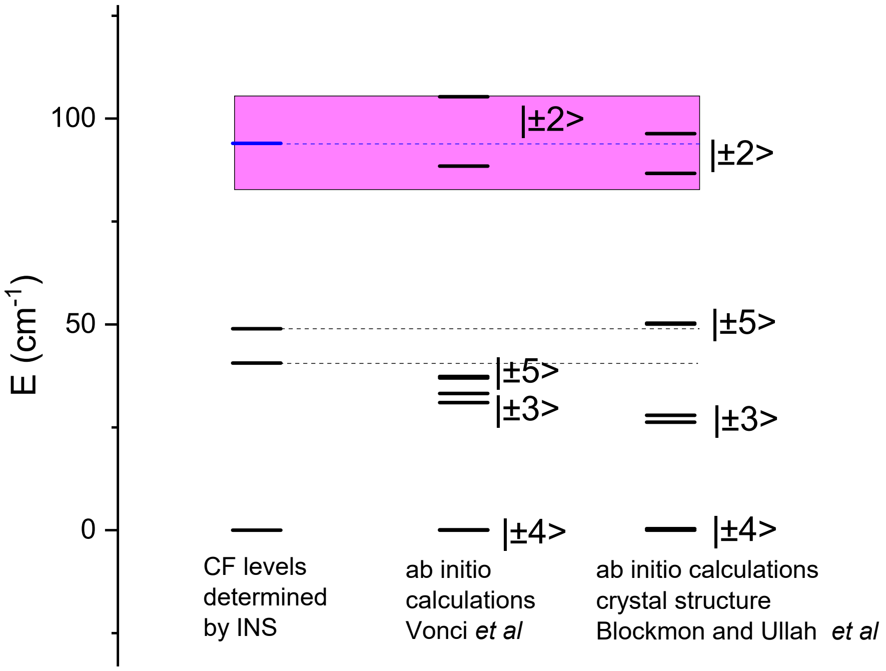

Figure S1: Left) energy level diagram of the experimentally determined crystal field transitions for $Na_9[Ho(W_5O_{18})_2]\cdot 35D_2O$. The two levels shown in black were observed by the direct observation of transitions between these levels. The third crystal field level (shown in blue) was determined using the vibronic coupling model described in the text. The pink highlighted region reflects the experimental uncertainty in the measurement. **Centre)** energy level diagram from ref 53, determined using CASSCF/RASSI. **Right)** Energy level diagram from ref 52 determined using CASSCF.

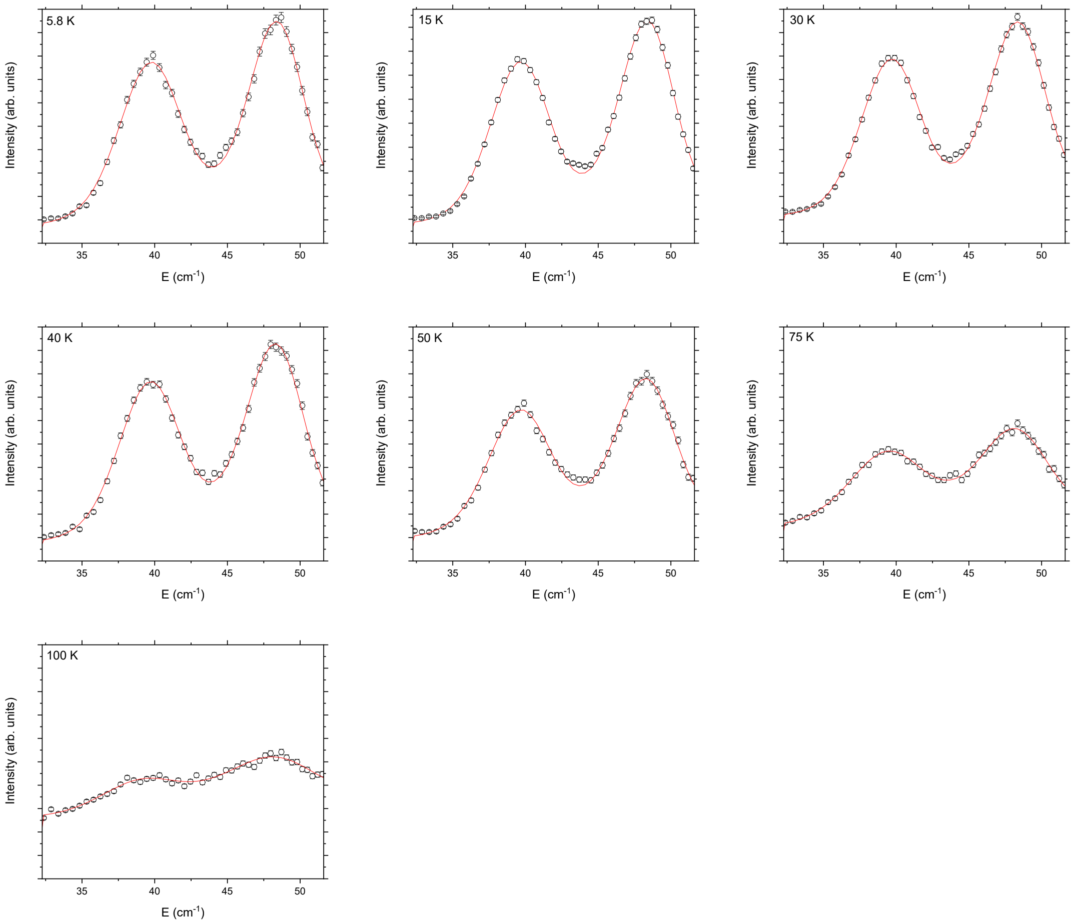

Figure S2: Fits of $S(\omega)$ obtained using 2.345 Å neutrons to model 1: two independent Lorentzian functions convoluted with the instrumental resolution function.

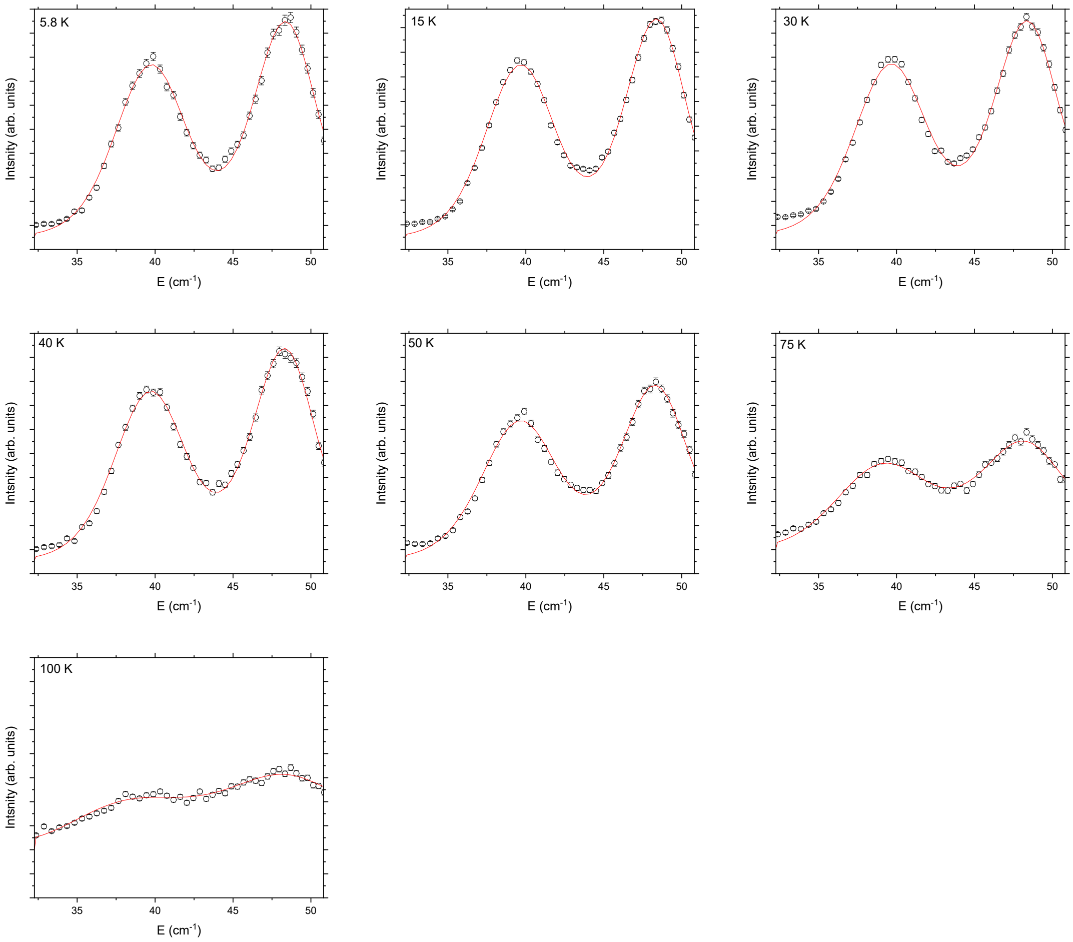

Figure S3: Fits of $S(\omega)$ obtained using 2.345 Å neutrons to model 2: two Lorentzian functions constrained to have the same peak width (independent position and height) convoluted with the instrumental resolution function.

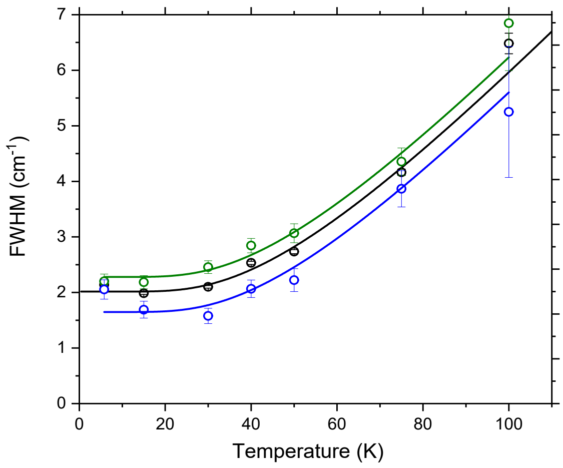

Figure S4. FWHM of ε_1 = 39.80 cm^{-1} (blue circles) and ε_2 = 48.28 cm^{-1} (green circles) allowed to vary independently (model 1) and fit to Equation 5, with Δ = 94.5 cm^{-1} and only Γ_0 allowed to vary (solid line, same colours as the data). The black circles are those obtained when the FWHM of ε_1 and ε_2 are constrained to be the same (model 2). This demonstrates that the magnetoelastic coupling parameter is the same for both excitations and that the only variation is in the residual, thus justifying the use of model 2 in the manuscript.

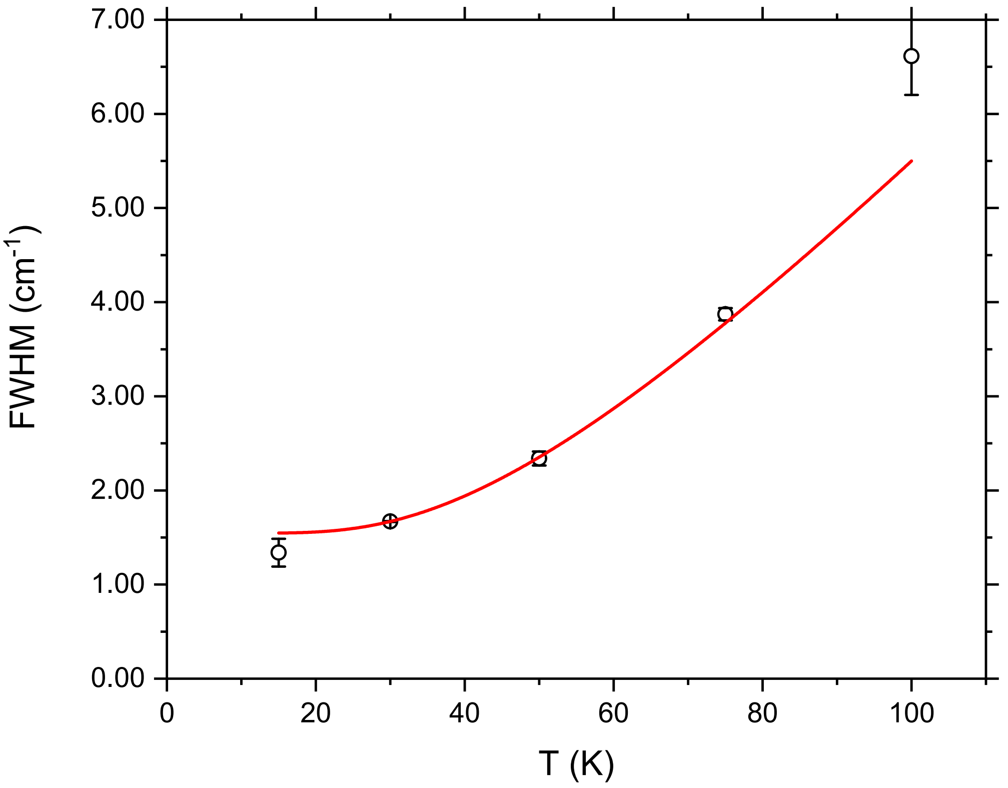

Figure S5. Results of fitting the neutron energy gain portion of the λ = 4.69 Å data. The fits were constrained to have a resolution FWHM of 3.15 cm^{-1}, which is the theoretical value for -43.55 cm^{-1} in this configuration (while the theoretical value for -47.59 cm^{-1} is 3.39 cm^{-1} and for 39.52 cm^{-1} is 2.82 cm^{-1}, with continuous variation between these two values). The fits were completed with the FWHM constrained to be the same width. Note that at 5.5 K, that neither crystal field excitation is thermally populated and thus are not observed. The low thermal population is also the reason for the comparatively large error at 15 K. Despite the lower resolution, the trend and values are clearly the same as those observed using neutron energy loss neutrons with 2.345 Å. The red line is a replication of the fit with Δ = 94.5 cm^{-1} and only Γ_0 allowed to vary

Definition of Phonon Density of States

In the current paper we refer to the generalised phonon density of states for both the inelastic neutron scattering data, as well as using the phonon density of states in the magnetoelastic coupling model. The two quantities are of course related. In the current manuscript we use:

$$g(Q,\omega) = \frac{\hbar\omega}{Q^2} S(Q,\omega)(1 - e^{\frac{-\hbar\omega}{kT}}) \qquad \text{S1}$$

This is the experimental neutron phonon density of states and this is determined from the measured scattering function S(Q,ω). It is note that this is not a normalised density of states, as the data have

been obtained without absolute units (which is the convention). The measured quantity $S(Q,\omega)$ also contains the instrumental constraints which include the kinematically allowed region and the resolution function of the spectrometer.

The quantity $Z(\omega)$ is used to describe the true vibrational density of states and is given by:

$$Z(\omega) = \frac{1}{3rN}\sum_{jq}\left|\varepsilon_{jq}(\Gamma)\right|^2\delta(\omega - \omega_{jq}) \qquad \text{S2}$$

Where *N* is the number of unit cells in the crystal, *j, q* are labels for the eigenstates, *r* is the number of atoms in the unit cell, and $\varepsilon_{jq}(\Gamma)$ are the symmetrised eigenvectors of the atoms.

The measured quantity $g(\omega)$ is used as an experimental verification of models of $Z(\omega)$, with caveats that it does not contain correction for both absolute normalisation and multiphonon effects. Convolution of $Z(\omega)$ with the instrument resolution function typically gives a good description of $g(\omega)$.

Pelican Resolution Function

The data presented in this paper rely on fits to both the inelastic scattering and quasielastic scattering convoluted with an appropriate instrument resolution function.

The resolution was determined in two ways.

Measurement of a vanadium standard is an effective measure of the instrument resolution at the elastic line.

The resolution function for a time-of-flight spectrometer such as Pelican is well known and has previously been described.[54] The resolution as a function of energy transfer is shown in Figure S6. This demonstrates that on the neutron energy loss side of the spectrum (positive energy transfers), that the vanadium resolution function is an effective approximation of the experimental resolution function.

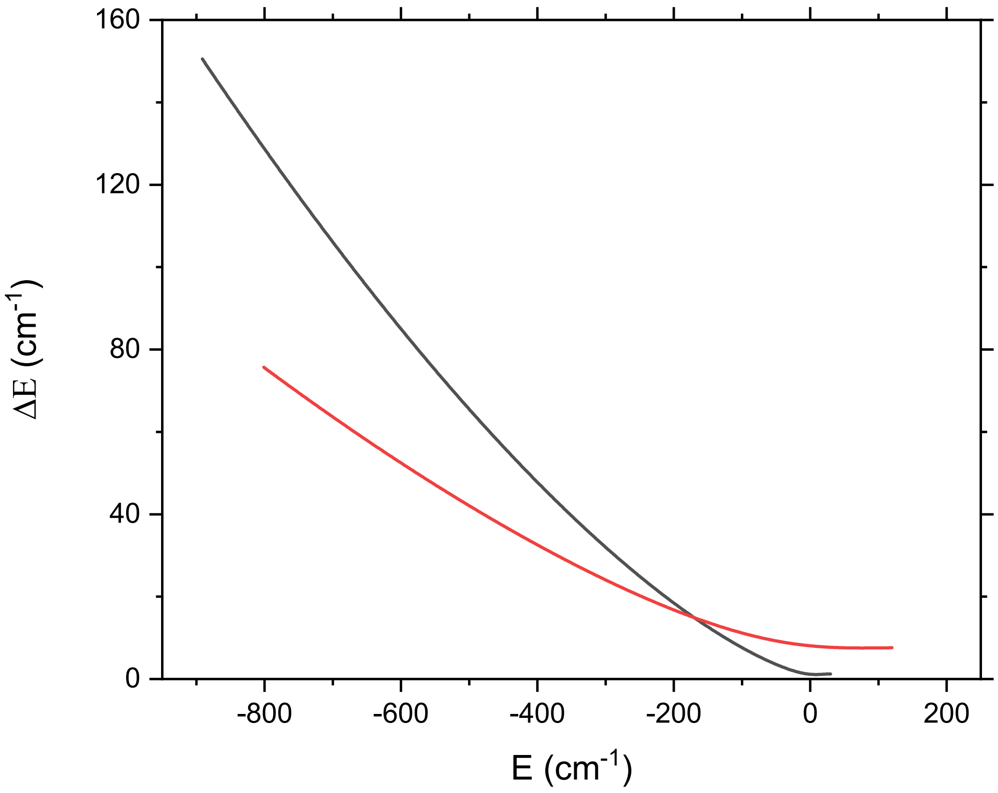

Figure S6. Calculated energy resolution *(ΔE)* for the time-of-flight spectrometer Pelican when using λ = 4.69 Å neutrons (black line) and λ/2 = 2.345 Å (red line)

Magnetoelastic Coupling Model

Here we introduce the magnetoelastic model first developed by Lovesey and Staub and reported in reference 58.

Central to the model is the introduction of a magnetoelastic interaction operator which is given by:

$$H^{'} = -\sum_{\Gamma_\nu} \zeta(\Gamma_\nu) u(\Gamma_\nu) Q(\Gamma_\nu) \quad \text{S3}$$

where $\zeta(\Gamma_\nu)$ is a magnetoelastic coupling parameter, $u(\Gamma_\nu)$ is a normal mode that transforms according to the representation Γ_ν, and $Q(\Gamma_\nu)$ is a quadrupole operator used to represent the 4*f* valence electrons of the Ho(III) ion.

To introduce the magnetoelastic coupling model, we must first introduce a CF model. In the case of Lovesey and Staub, a parameterised CF model is used, though in the case of $Na_9[Ho(W_5O_{18})_2]\cdot 35D_2O$ we have previously reported the inelastic neutron scattering and corresponding ab initio calculations ref 53.

The key step for looking at magnetoelastic coupling is to introduce a three-state model. In the first instance we will start with a model that contains a ground state, a first excited state at energy ε and a second excited state at energy Δ from the elastic line. Sticking with the notation of Lovesey and Staub, these are labelled *a*, *b* and γ. The magnetoelastic interaction can couple the two lowest lying states *a* and *b* to the second excited state γ

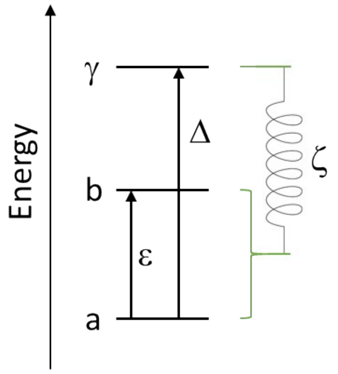

Figure S7: Schematic showing the simple energy level diagram used to construct the magnetoelastic coupling model

The next step is fully described in reference 58, but the CF states and the interaction between states is mapped to an appropriate Hamiltonian, including the term described in S3, for the three-state model (not the entire manifold of crystal field levels).

In doing so lattice displacements are introduced into the Hamiltonian. These are assumed to be harmonic. Ultimately this leads to the incorporation of the phonon density of states in the model. This is given by equation S2 above.

Ultimately using second order perturbation theory it is possible to write the relaxation rate observed by inelastic neutron scattering for this three state model as:

$$\Gamma(T) = \left(\frac{3\pi r}{M}\right)\left\{\frac{1}{\varepsilon} cotanh\left(\frac{\varepsilon}{2T}\right)\left[(\zeta\langle a\,|\,Q\,|\,b\rangle)^2 Z(\varepsilon)\right]_{\Gamma} + (\zeta\langle b\,|\,Q\,|\,\gamma\rangle)^2_{\Gamma'}\frac{n(\Delta-\varepsilon)}{(\Delta-\varepsilon)}[Z(\Delta-\varepsilon) + Z(\text{[illegible]}\right. \quad \text{S4}$$

Here the function $\Gamma(T)$ is the relaxation rate, while the subscript Γ, Γ' and Γ'' indicate that ζ, *Q* and $Z(\omega)$ depend on a symmetry representation, with no requirement that these are the same for three terms above. This can be further simplified in the case of $Na_9[Ho(W_5O_{18})_2]\cdot 35D_2O$, where the central lanthanoid ion has a point group symmetry of C_S and thus can be described by a single irreducible representation.

In equation S4, the phonon density of states contributes at three energies, ε, Δ and Δ-ε.

At this point we can now make some simplifications based on the observed CF levels of $Na_9[Ho(W_5O_{18})_2]\cdot 35D_2O$.

Initially let us consider the three states with energies shown in table S1.:

Table S1 Energy levels assuming the three lowest crystal field levels are required.

	a	***b***	γ	$\varDelta$	$\varDelta$-ε
E	0	39.80	48.28	48.28	8.48
m_J	|±4>	|±3>	|±5>		

In this case we know that the phonon density of states Z(Δ) and Z(Δ-ε) are both approximately zero. This can be demonstrated in two ways. Firstly, the fits to the CF excitations shown in figure S3 included no background contribution. Thus, the intensity is fully accounted for by the crystal field excitation. As such we can approximate that both Z(Δ) and Z(Δ-ε) are small.

Secondly the lattice dynamics calculations do not predict any vibrational spectra below 99.6 cm^{-1} while the FIR spectra have a lowest measured peak at 68.4 cm^{-1}. In this case, all the terms in equation S4 are negligible.

We next consider a different three states listed in table S2:

Table S2. Energy levels for the second proposed set of crystal fields for the magnetoelastic model

	a	***b***	γ	$\varDelta$	$\varDelta$-ε
E	0	39.80	94.5	94.5	54.7
m_J	|±4>	|±3>	|±2>		

In this case Z(Δ-ε) also lies in the region of the CF excitation. It also lies below the energy calculated by the vibrational spectra to show intensity and is below the measured peak in the vibrational spectra measured by FIR.

Thus equation S4 can be simplified to

$$\Gamma(T) = \Gamma_0 + \frac{d\zeta^2 Z(\Delta)}{2\Delta(e^{\frac{\Delta}{kT}} - 1)} \qquad \text{S5}$$

Here we have introduced the residual Γ_0, which can occur as a result of relaxation via other pathways. This is possible within equation S4, as while *Z*(*Δ*-*ε*) is small, it will likely not be zero. While the value of Γ_0 is included as a constant, it is worth highlighting that this depends on ε and the nature of the states a and b, so this is expected to be different for each excitation, as observed in Figure S4. Note the factor of two occurs as we measured the full width at half maximum (FWHM), while the lifetime, Γ(T) corresponds to the Half width at half maximum (HWHM).

The constant d includes the matrix elements from the previous expression. These are in effect a scaling factor. As we are using arbitrary units it is not possible to extract these values (or the strength of the magnetoelastic coupling) from the current dataset.

Finally we consider the energy levels shown in table S3:

Table S3. Energy levels for the third proposed set of crystal fields for the magnetoelastic model

	a	***B***	γ	Δ	Δ-ε
E	0	48.28	94.5	94.5	46.22
m_J	|±4>	|±5>	|±2>		

Again this corresponds to the case that the first two terms in equation S4 can be neglected and the temperature dependence described by eqn S5.

Higher states. It is worth stressing here that the value of delta of 94.5 cm^{-1} was determined as a fitting parameter. At high temperatures when T>>Δ this will give $\Gamma(T) \propto T$. This comes from the temperature dependent behaviour of n(Δ). However, the temperature at which this occurs is directly dependent on Δ. This is visualised in the top panel of figure 7, where the same magnetoelastic coupling constant used to the other crystal field states (different Δ values) clearly does not correspond to the observed FWHM broadening.

It should also be highlighted that this temperature dependence only describes the relaxation via a magnetoelastic coupling model. Other relaxation pathways (eg Raman) may be possible in this compound, however these are not observed as a temperature dependence to the FWHM.
